# Access to female contraceptives by Rohingya refugees, Bangladesh

**DOI:** 10.2471/BLT.20.269779

**Published:** 2021-01-07

**Authors:** Md Nuruzzaman Khan, M Mofizul Islam, Md Mashiur Rahman, Md Mostafizur Rahman

**Affiliations:** a Department of Population Sciences, Jatiya Kabi Kazi Nazrul Islam University, Trishal, Mymensingh-2220, Bangladesh.; b Department of Public Health, La Trobe University, Melbourne, Australia.; c Department of Sociology, University of Rajshahi, Rajshahi, Bangladesh.; d Department of Population Science and Human Resource Development, University of Rajshahi, Rajshahi, Bangladesh.

## Abstract

**Objective:**

To determine the prevalence of the use of contraceptives among female Rohingya refugees in Bangladesh and its associated factors.

**Methods:**

We conducted our cross-sectional survey at the Kutupalong refugee facility located in Cox’s Bazar in November 2019. We used univariable and multivariable logistic regression models to determine the association between the use of contraceptives and our various predictor variables, including women’s age, age at first marriage, education level and employment status. We also considered factors such as whether previous pregnancies were planned or unplanned, and the occurrence of non-consensual sex with husbands.

**Findings:**

We found that 50.91% (251/493) of the survey participants used contraceptives, and that injection (169/251; 67.33%) and oral contraceptives (75/251; 29.88%) were the predominant modes. Of the women who did not use contraceptives, the main reasons were reported as disapproval by husbands (118/242; 48.76%), actively seeking a pregnancy (42/242; 17.36%) and religious beliefs (37/242; 15.29%). An increased likelihood of using contraceptives was found to be positively associated with women’s employment outside their households (odds ratio, OR: 3.11; 95% confidence interval, CI: 1.69–6.11) and the presence of a health-care centre in the camp (OR: 3.92; 95% CI: 2.01–7.67). Women who reported an unplanned pregnancy during the previous 2 years were less likely to use contraceptives (OR: 0.02; 95% CI: 0.01–0.05).

**Conclusion:**

To increase the acceptance and use of contraceptives, we recommend programmes targeted at women of reproductive age and their husbands, religious and community leaders, and providers of family planning and child and maternal health-care services.

## Résumé

**Objectif:**

Déterminer la fréquence d'utilisation de contraceptifs chez les femmes Rohingya réfugiées au Bangladesh, ainsi que les facteurs qui y sont associés.

**Méthodes:**

Nous avons mené notre enquête transversale au sein du camp de réfugiés de Kutupalong, dans le district de Cox's Bazar, en novembre 2019. Nous avons employé des modèles de régression logistique univariée et multivariée pour identifier le lien entre le recours aux contraceptifs et nos différentes variables prédictives: âge des femmes, âge lors du premier mariage, niveau d'éducation et situation professionnelle. Enfin, nous avons tenu compte de divers facteurs, notamment le fait que les précédentes grossesses aient été planifiées ou pas, et l'existence de rapports sexuels non consentis avec le mari.

**Résultats:**

Nous avons constaté que 50,91% (251/493) des participantes à l'enquête prenaient des contraceptifs, et que les contraceptifs injectables (169/251; 67,33%) et oraux (75/251; 29,88%) étaient les plus répandus. Chez les femmes qui ne prenaient aucun contraceptif, les principales raisons invoquées étaient la désapprobation du mari (118/242; 48,76%), la volonté de concevoir un enfant (42/242; 17,36%) et les croyances religieuses (37/242; 15,29%). La probabilité de recourir à des contraceptifs augmentait lorsque les femmes exerçaient un emploi hors de leur foyer (odds ratio, OR: 3,11; intervalle de confiance de 95%, IC: 1,69–6,11) et lorsqu'un centre médical était présent dans le camp (OR: 3,92; IC de 95%: 2,01-7,67). Les femmes ayant signalé une grossesse non planifiée au cours des deux dernières années étaient moins susceptibles d'utiliser des contraceptifs (OR: 0,02; IC de 95%: 0,01-0,05).

**Conclusion:**

Afin que les contraceptifs soient mieux acceptés et employés, nous recommandons d'opter pour des programmes ciblant les femmes en âge de procréer et leur mari, les autorités religieuses et responsables communautaires, ainsi que les prestataires de services de planification familiale, de santé infantile et de santé maternelle.

## Resumen

**Objetivo:**

Determinar la prevalencia del uso de anticonceptivos entre las refugiadas rohingya de Bangladesh y sus factores conexos.

**Métodos:**

Realizamos nuestra encuesta transversal en el centro de refugiados de Kutupalong en Cox's Bazar en noviembre de 2019. Utilizamos modelos de regresión logística univariables y multivariables para determinar la asociación entre el uso de anticonceptivos y nuestras diversas variables de predicción, incluyendo la edad de las mujeres, la edad del primer matrimonio, el nivel de educación y la situación laboral. También consideramos factores tales como si los embarazos anteriores fueron planificados o no, y la existencia del acto sexual no consensuado entre el matrimonio.

**Resultados:**

Comprobamos que el 50,91% (251/493) de los participantes en la encuesta utilizaban anticonceptivos, y que la modalidad predominante era los anticonceptivos inyectados (169/251; 67,33%) y orales (75/251; 29,88%). De las mujeres que no utilizaron anticonceptivos, las principales razones fueron la desaprobación de los maridos (118/242; 48,76%), la búsqueda activa de un embarazo (42/242; 17,36%) y las creencias religiosas (37/242; 15,29%). Descubrimos que la mayor probabilidad de utilizar anticonceptivos estaba positivamente asociada con el empleo de las mujeres fuera de sus hogares (proporción de probabilidades, RM: 3,11; intervalo de confianza del 95%, IC: 1,69-6,11) y con la presencia de un centro de salud en el campamento (RM: 3,92; IC del 95%: 2,01-7,67). Las mujeres que informaron de un embarazo no planificado durante los dos años anteriores tenían menos probabilidades de utilizar anticonceptivos (RM: 0,02; IC del 95%: 0,01-0,05).

**Conclusión:**

Para aumentar la aceptación y el uso de los anticonceptivos, recomendamos programas dirigidos a las mujeres en edad de procrear y a sus maridos, a los dirigentes religiosos y comunitarios, así como a los proveedores de servicios de planificación familiar y de atención sanitaria maternoinfantil.

## ملخص

الغرض تحديد مدى انتشار استخدام موانع الحمل بين لاجئات الروهينجا في بنغلاديش، والعوامل المرتبطة بذلك.

الطريقة أجرينا مسحًا متعدد القطاعات في مرفق كوتوبالونغ للاجئين الواقع في كوكس بازار في نوفمبر/تشرين ثان 2019. استخدمنا نماذج التحوف اللوجيستي أحادية المتغير ومتعددة المتغيرات لتحديد الارتباط بين استخدام موانع الحمل ومتغيراتنا التنبؤية المتنوعة، بما في ذلك عمر المرأة، والعمر عند أول زواج، والمستوى التعليمي، والوضع الوظيفي. كما أخذنا في الاعتبار أيضًا عوامل مثل ما إذا كانت حالات الحمل السابقة مخطط لها أم غير مخطط لها، وممارسة الجنس مع الأزواج من غير موافقة.

النتائج وجدنا أن 50.91% (251/493) من المشاركين في المسح استخدموا موانع الحمل، وأن الحقن (169/251؛ 67.33%)، ووسائل منع الحمل عن طريق الفم (75/251؛ 29.88%) كانت هي السائدة. وفقًا للنساء اللاتي لم يستخدمن وسائل منع الحمل، كانت الأسباب الرئيسية المبلغ عنها رفض الأزواج (118/242؛ 48.76%)، والسعي بنشاط للحمل (42/242؛ 17.36%)، والمعتقدات الدينية (37/242 ؛ 15.29٪). تم اكتشاف أن الاحتمالية المتزايدة لاستخدام وسائل موانع الحمل مرتبطة بشكل إيجابي بتوظيف النساء خارج أسرهن (نسبة الاحتمالات: 3.11؛ فاصل الثقة 95%: 1.69 إلى 6.11) ووجود مركز للرعاية الصحية في المخيم (نسبة الاحتمالات: 3.92؛ ‏بفاصل ثقة 95%: 2.01–7.67). كانت النساء اللاتي أبلغن عن حمل غير مخطط له خلال العامين الماضيين أقل احتمالية لاستخدام وسائل مانع الحمل (نسبة الاحتمالات: 0.02؛ ‏بفاصل ثقة 95%: 0.01–0.05).

الاستنتاج لزيادة قبول واستخدام وسائل موانع الحمل، نحن نوصي بالبرامج التي تستهدف النساء في سن الإنجاب وأزواجهن، والزعماء الدينيين والمجتمعيين، ومقدمي خدمات تنظيم الأسرة وخدمات رعاية صحة الطفل والأم.

## 摘要

**目的:**

确定孟加拉国罗兴亚女性难民避孕药具的使用情况及其相关因素。

**方法:**

2019 年 11 月，我们在考克斯巴扎尔的库图帕朗难民机构开展了横向调查。我们使用单变量和多变量逻辑回归模型，来确定避孕药具的使用与各种预测变量之间的关联，包括妇女的年龄、初婚年龄、受教育程度和就业状况。我们还考虑了一些因素，譬如，之前怀孕是有计划的还是无计划的，以及是否与丈夫发生了非自愿的性行为。

**结果:**

我们发现，有 50.91% (251/493) 的调查参与者使用了避孕药具，其中避孕针剂 (169/251; 67.33%) 和口服避孕药 (75/251; 29.88%) 是主要避孕方式。据报告，有些妇女没有使用避孕药具的主要原因是丈夫不赞成 (118/242; 48.76%)、积极寻求怀孕 (42/242; 17.36%) 和受宗教信仰影响 (37/242; 15.29%)。我们发现，使用避孕药具的可能性与妇女在家庭之外的就业情况（优势比，OR: 3.11; 95％ 置信区间，CI: 1.69–6.11）以及营地是否设有保健中心 (OR: 3.92; 95％ CI: 2.01-7.67) 呈正相关。在过去两年中，报告计划外怀孕的妇女使用避孕药具的可能性较小 (OR: 0.02; 95% CI: 0.01–0.05)。

**结论:**

为了提高避孕药具的接受率和使用率，我们建议针对育龄妇女及其丈夫、宗教和社区领袖，以及计划生育和妇幼保健服务提供者制定相关方案。

## Резюме

**Цель:**

Определить распространенность применения противозачаточных средств среди беженок рохинджа в Бангладеш и связанные с этим факторы.

**Методы:**

В ноябре 2019 года авторы провели перекрестно-групповое исследование в центре для беженцев Кутупалонг, расположенном в Кокс-Базаре. Авторы использовали однофакторные и многофакторные модели логистической регрессии, чтобы определить связь между применением противозачаточных средств и различными прогностическими переменными, включая возраст женщин, возраст вступления в первый брак, уровень образования и статус занятости. Мы также рассмотрели такие факторы, как запланированные или незапланированные предыдущие беременности, а также наличие секса с мужьями без обоюдного согласия.

**Результаты:**

Мы установили, что 50,91% (251/493) участников опроса применяли противозачаточные средства и что преобладающим методом были инъекции (169/251; 67,33%) и оральные контрацептивы (75/251; 29,88%). Среди женщин, которые не пользовались противозачаточными средствами, основными причинами были неодобрение со стороны мужей (118/242; 48,76%), активное стремление забеременеть (42/242; 17,36%) и религиозные убеждения (37/242; 15,29%). Было обнаружено, что повышенная вероятность применения противозачаточных средств положительно связана с занятостью женщин за пределами своей семьи (отношение шансов, ОШ: 3,11; 95%-й ДИ: 1,69–6,11) и наличием медицинского учреждения в лагере (ОШ: 3,92; 95%-й ДИ: 2,01–7,67). Женщины, сообщившие о незапланированной беременности в течение предыдущих 2 лет, реже применяли противозачаточные средства (ОШ: 0,02; 95%-й ДИ: 0,01–0,05).

**Вывод:**

С целью повышения уровня принятия и применения противозачаточных средств рекомендуется проведение программ, ориентированных на женщин репродуктивного возраста и их мужей, религиозных и местных общественных деятелей, а также поставщиков услуг по планированию семьи и охране здоровья матери и ребенка.

## Introduction

The women of forcibly displaced populations worldwide face a substantial degree of adverse health outcomes as a result of arranged or forced marriage when young, unprotected sexual intercourse and limited access to health-care services. These situations result in large numbers of unplanned pregnancies and unsafe abortions, and the associated complications.^1^^,^^2^ Contraception is highly recommended to prevent such adverse outcomes, and is an essential component of basic sexual and reproductive health responses in humanitarian emergencies.^1^ Such health-care services include emergency contraception, long- and short-term contraceptive methods, menstrual regulation services and post-abortion care.^3^ The provision of contraception services also offers women many other health-improving associated benefits, such as the autonomy to determine the number and timing of their children, and to access opportunities related to their education and livelihoods. Awareness of contraception can also empower families to manage scarce resources and reduce the burden of maternal and child malnutrition.^4^ To achieve the third sustainable development goal, that is ensuring healthy lives and promoting well-being for all at all ages, by 2030, the World Health Organization prioritizes contraception services in refugee settings to improve maternal health.^5^^,^^6^

The Rohingya people living in refugee camps in Bangladesh began to migrate from Myanmar in 1970. However, the largest numbers arrived in 2017 following a widespread operation by Myanmar’s military that was later declared genocide in a hearing at the International Court of Justice.^7^ While living in Myanmar, the Rohingya people have historically been denied citizenship and basic human rights, severely restricting their access to education and health services. These restrictions have had a substantial impact on their knowledge of the use of contraception and family planning,^8^ meaning that overall fertility and adverse health outcomes related to pregnancy, including maternal and child mortality, were extremely high.^9^ The situation has remained essentially unchanged following their forced migration to Bangladesh.^1^^,^^3^

The Ministry of Health and Family Welfare of the Government of Bangladesh, along with around 150 national and international development partners such as nongovernmental organizations (NGOs), United Nations agencies and other international agencies (e.g. Cooperative for Assistance and Relief Everywhere International and Save the Children), are now offering health and family planning services in the refugee camps.^10^^,^^11^ Under this initiative, family planning health-care workers visit women’s homes at a recommended frequency of every 2 weeks to provide family planning information and counselling, and/or dispense contraceptives.^3^^,^^12^ Medical clinics and dispensaries with facilities for minor surgeries are also provided within refugee camps, and some over-the-counter drugs are available from shops and groceries around the camps accessible to both local residents and refugees. Patients requiring secondary and tertiary care are transferred to local government medical college hospitals in either Cox’s Bazar or Chattogram. 

However, little is known regarding whether such initiatives have made any impact on the use of contraceptives among female refugees.^8^^,^^13^ We therefore used a cross-sectional survey to explore the prevalence of contraceptive use and to determine the factors associated with the use, or barriers to the use, of contraceptives among female Rohingya refugees in Bangladesh.

## Methods

### Study setting

We conducted our study at the Kutupalong refugee makeshift facility located at Ukhiya, Cox’s Bazar, Bangladesh. This facility was established in 1991, and most of the Rohingya refugees have resided there since they departed Myanmar in 2017. Around 700 000 Rohingya refugees and their temporary homes are crowded into an area of only 13 km^2^, making it the world’s largest refugee settlement in terms of both size and population density. Administratively, the facility is divided into 34 camps comprising a total of 208 blocks; on average, each block supports 892 households.^14^

### Study design

We conducted our cross-sectional survey during the second week of November 2019. We first randomly selected two blocks from each of four randomly selected camps. Women who had married or given birth during the previous 2 years were invited to participate in the survey, and we endeavoured to interview as many eligible women as possible within the selected eight blocks during the week of our survey. To identify all eligible participants for whom pregnancy was an option, we excluded women who were already pregnant, and women who had not been sexually active during the month before the survey. Six female interviewers conducted the interview, all of whom had completed a university or equivalent degree and had experience of interviewing Rohingya people. 

The interviewers conducted the survey using a structured questionnaire. We developed the primary version of our questionnaire by incorporating relevant questions from the Demographic and Health Survey.^15^^,^^16^ Before the survey, we piloted our questionnaire with 15 Rohingya women to ensure its appropriateness. We addressed some inconsistencies at this stage, mostly in the answer options to some questions and in terms used to identify health-care providers in the camps. 

### Outcome variable

We derived data on women’s use of contraceptives from the question: “Are you currently doing something or using any methods to delay or avoid getting pregnant?” The response options to this question were “yes” or “no.” Women who responded “yes” were then asked to identify the methods they use from the following list: contraceptive injection, oral contraceptive pill, implant, female sterilization, intrauterine device, periodic abstinence, male sterilization, condom or withdrawal. We also provided a free-text response option for use if their contraceptive method was not included in this list. Where women reported the use of multiple contraceptive methods, the method used most frequently was identified as the usual method of contraception. Women who responded “no” were asked about their reasons for not using contraception.

### Predictor variables

We identified potential predictor variables from previously published literature on contraception and family planning in refugee settings,^4^^,^^17^^,^^18^ including the continuous variables women’s age, age at first marriage and number of live births, and the categorical variables education level and employment status. Regarding employment status, Rohingya refugees can engage in work within the camp under the cash-for-work scheme run by some NGOs, albeit on a limited scale.^19^^,^^20^

We asked whether each woman had had a live birth within the previous 2 years and, if yes, whether this pregnancy was planned or unplanned. We also asked whether women had non-consensual sexual intercourse with their husbands, by posing the question “Does your husband force you to have sex without your consent?” If the answer was positive we asked whether such incident was occasionally or frequently (We note that WHO defines all non-consensual sex as rape). We included husband’s age, education level and employment status as possible predictor variables. Finally, we considered the availability of health-care services in the camp as a dichotomous predictor variable.

### Statistical analysis

We used descriptive statistics to characterize the demographic profile of women included in our analysis. We used univariable and multivariable logistic regression models to determine the association between the outcome variable and predictor variables. We included all available predictor variables associated with the use of contraceptives in our multivariable model. We performed all statistical analyses using the software Stata version 15.1 (StataCorp, College Station, United States of America), and report all associations as odds ratios (OR) with 95% confidence intervals (CIs). 

### Ethics

We received ethical approval for this survey from the Institute of Biological Science at Rajshahi University (approval number: 123/320/IAMEBBC/IBSc), Bangladesh. We obtained verbal consent from all participants before interviews. The interviewers endeavoured to ensure the confidentiality of the selected respondents by conducting private interviews either in the participants’ houses or outside, excluding others. To ensure privacy was obtained, interviewers occasionally visited some houses more than once.

## Results

A total of 508 women were interviewed, and our final sample of eligible participants consisted of 493 women.

The women who participated in our survey had a mean age of 23.34 years (standard deviation: 5.14) and had a mean of 2.55 children (Table 1). We found that almost three quarters (367/493; 74.44%) had received no formal education, and around two thirds (329/493; 66.73%) reported that their husbands had received no formal education (Table 2). Over three quarters of the women (376/493; 76.27%) reported that they did not work outside of their homes. Most of the women reported that their husbands were either day labourers (226/493; 45.84%) or were engaged in voluntary jobs and services (176/493; 35.70%).

**Table 1:** Characteristics of female Rohingya refugees included in study of contraceptive use, Bangladesh, November 2019

Characteristic	Mean (SD)
**Age (years)**	
**Women **	23.34 (5.14)
Women currently using contraceptives	23.24 (5.28)
At which first married	15.77 (4.09)
Women not currently using contraceptives	23.45 (5.00)
At which first married	16.29 (2.84)
**Husbands**	28.74 (7.88)
Of women currently using contraceptives	28.56 (7.20)
Of women not currently using contraceptives	28.94 (8.53)
**No. of live births**	2.55 (2.12)
To women currently using contraceptives	2.45 (2.03)
To women not currently using contraceptives	2.66 (2.20)

SD: standard deviation.

**Table 2:** Sociodemographic properties of and contraceptive use among female Rohingya refugees, Bangladesh, November 2019

Variable	No. (%)
**Women’s education level (*n* = 493)**	
No formal education^a^	367 (74.44)
Some formal education	126 (25.56)
**Women’s employment status (*n* = 493)**	
Only household work	376 (76.27)
Engaged in additional work	117 (23.73)
**Husband’s education level (*n* = 493)**	
No formal education^a^	329 (66.73)
Some formal education	164 (33.27)
**Husband’s employment status (*n* = 493)**	
Unemployed	91 (18.46)
Day labourer	226 (45.84)
Other voluntary work	176 (35.70)
**Contraceptive use (*n* = 493)**	
Yes	251 (50.91)
No	242 (49.09)
**If yes, type (*n* = 251)**	
Contraceptive injection (Depo-Provera®)	169 (67.33)
Oral contraceptive pill	75 (29.88)
Implant	3 (1.20)
Female sterilization	2 (0.80)
Intrauterine device	1 (0.40)
Periodic abstinence	1 (0.40)
Male sterilization, condom or withdrawal	0 (0.00)
**If no, reason (*n* = 242)**	
Disapproval by husband	118 (48.76)
Pregnancy desired	42 (17.36)
Religious beliefs	37 (15.29)
Unaware of good methods of contraception	13 (5.37)
Irregular sex	11 (4.55)
Menopause	8 (3.31)
Dislike of family planning	6 (2.48)
Other^b^	7 (2.89)

^a^ Attended no formal educational institutions, although some had attended institutions such as *madrasah* (a mainly religious and basic education provider).

^b^ Including social pressure, being asked to re-attend health-care facility at a later data to collect contraceptives, no opportunity, no need and having a stomach ulcer.

We found that slightly over half of the women (251/493; 50.91%) were using contraceptives at the time of our survey (Table 2); the largest proportion of these women reported use of the contraceptive injection (169/251; 67.33%), followed by the oral contraceptive pill (75/251; 29.88%). The numbers reporting use of an implant (3/251; 1.20%) or having undergone sterilization (2/251; 0.80%) were very small. 

We observed that the most common reason for not using contraceptives (242/493; 49.09%) was disapproval by the women’s husbands (118/242; 48.76%), followed by actually desiring a pregnancy (42/242; 17.36%) and religious beliefs (37/242; 15.29%; Table 2). 

Although the recommended frequency of home visits by health-care workers is once every 2 weeks, only 10.95% (54/493) of the women reported receiving such frequent visits during the 3 months immediately before the survey. More than half of the women (255/493; 51.72%) reported no visits, and around one quarter (121/493; 24.54%) received only 1–3 visits (Table 3). Women who received a visit that included both a family planning consultation and provision of contraceptives (57/493; 11.56%) were more likely to use contraceptives (41/57, 71.93%) than women who either (i) participated in a consultation, but were not supplied with contraceptives (75/144; 52.08%) or (ii) were supplied with contraceptives, but did not receive a consultation (23/37; 62.16%). Of the women who received a single visit by a family planning health-care worker, 46.15% (12/26) reported using contraceptives; this proportion increased to 57.14% (20/35) among women who received five visits (Table 3).

**Table 3:** Nature and frequency of family planning personnel visits received by female Rohingya refugees, Bangladesh, November 2019

Family planning personnel visit	No. (%)		
	Total (*n* = 493)	Women using contraceptives (*n* = 251)	Women not using contraceptives (*n* = 242)
**Whether visit occurred and nature of visit**			
No	255 (51.72)	112 (44.62)	143 (59.09)
Yes, and discussed family planning	144 (29.21)	75 (29.88)	69 (28.51)
Yes, and supplied contraceptives	37 (7.51)	23 (9.16)	14 (5.79)
Yes, and discussed family planning and supplied contraceptives	57 (11.56)	41 (16.33)	16 (6.61)
**No. of visits^a^**			
0	255 (51.72)	112 (44.62)	143 (59.09)
1	26 (5.27)	12 (4.78)	14 (5.79)
2	45 (9.13)	23 (9.16)	22 (9.09)
3	50 (10.14)	25 (9.96)	25 (10.33)
4	28 (5.68)	16 (6.37)	12 (4.96)
5	35 (7.10)	20 (7.97)	15 (6.20)
6	29 (5.88)	18 (7.17)	11 (4.55)
≥ 7	25 (5.07)	25 (9.96)	0 (0.00)

**^a^** No. of visits during three months immediately before the survey.

The use of contraceptives by women varied with several sociodemographic properties (Table 4). We noted that the level of education received by either the women or their husbands was not associated with the women’s use of contraceptives. However, the 117 women engaged in additional work outside their household were more likely to report the use of contraceptives (71/117; 60.68%) compared with the 376 women who did not work outside their home (180/376; 47.87%). A large proportion of the 244 women whose last pregnancy was planned reported using contraceptives at the time of the survey (190/244; 77.87%). We also recorded a greater use of contraceptives among women who: (i) reported no non-consensual sex with their husbands in the year before the survey (113/213; 53.05%) compared with women who reported occasional or frequent non-consensual sex (138/280; 49.29%); and (ii) resided in a camp with a mobile health-care centre (213/409; 52.08%) compared with women whose camp did not have such facilities (38/84; 45.24%).

**Table 4:** Differences in sociodemographic properties between female Rohingya refugees using and not using contraceptives, Bangladesh, November 2019

Characteristic	No. (%)	
	Women using contraceptives (*n* = 251)	Women not using contraceptives (*n* = 242)
**Women’s education level**		
No formal education^a^	189 (75.30)	178 (73.55)
Formal education	62 (24.70)	64 (26.45)
**Women’s employment status**		
Only household work	180 (71.71)	196 (80.99)
Engaged in additional work	71 (28.29)	46 (19.01)
**Husband’s education level**		
No formal education^a^	171 (68.13)	158 (65.29)
Formal education	80 (31.87)	84 (34.71)
**Husband’s employment status**		
Unemployed	47 (18.73)	44 (18.18)
Day labourer	120 (47.81)	106 (43.80)
Other voluntary work	84 (33.47)	92 (38.02)
**Live birth within past 2 years**		
Yes, planned	190 (75.70)	54 (22.31)
No	47 (18.73)	26 (10.74)
Yes, unplanned	14 (5.58)	162 (66.94)
**Had non-consensual sexual intercourse with husband in past 12 months**		
Never	113 (45.02)	100 (41.32)
Occasionally	100 (39.84)	107 (44.21)
Frequently	38 (15.14)	35 (14.46)
**Availability of health-care services in the camp**		
No health-care centre, but field workers visit home	38 (15.14)	46 (19.01)
Yes	213 (84.86)	196 (80.99)

^a^ Attended no formal educational institutions, although some had attended institutions such as *madrasah* (a mainly religious and basic education provider).

From our multivariable regression model, we observed that five variables were significantly associated with the use of contraceptives: two positively and three negatively (Table 5). We note that the positively influencing factors included women’s employment outside their households (OR: 3.11; 95% CI: 1.69–6.11) and the existence of a health-care centre in their camp (OR: 3.92; 95% CI: 2.01–7.67). Women whose most recent pregnancy was unplanned were significantly less likely (OR: 0.02; 95% CI: 0.01–0.05) to report the use of contraceptives than women whose last pregnancy was planned. We noted a 13% reduction (OR: 0.87; 95% CI: 0.76–0.98) in use of contraceptives for every 1 year decrease in age at first marriage.

**Table 5:** Factors affecting likelihood of use of contraceptives by female Rohingya refugees, Bangladesh, November 2019

Sociodemographic property	OR (95% CI)	
	Univariable model	Multivariable model
**Women’s age**	0.99 (0.96–1.03)	1.00 (0.89–1.12)
**Women’s education**		
No formal education^a^	Ref.	Ref.
Formal education	0.91 (0.61–1.37)	1.24 (0.67–2.30)
**Women’s age at first marriage**	0.96 (0.91–1.01)	0.87 (0.76–0.98)
**Previous live births**	0.95 (0.88–1.04)	0.92 (0.73–1.17)
**Women’s employment status **		
Only household work	Ref.	Ref.
Engaged in additional work	1.68 (1.10–2.56)	3.11 (1.69–6.11)
**Husband’s age**	0.99 (0.97–1.02)	1.00 (0.95–1.06)
**Husband’s education level**		
No formal education^a^	Ref.	Ref.
Formal education	0.88 (0.60–1.28)	1.03 (0.59–1.79)
**Husband’s employment status**		
Unemployed	Ref.	Ref.
Day labourer	1.06 (0.65–1.72)	1.09 (0.53–2.23)
Other voluntary work	0.85 (0.52–1.42)	1.06 (0.50–2.24)
**Live birth within past 2 years**		
Yes, planned	Ref.	Ref.
No	0.51 (0.29–0.91)	0.39 (0.17–0.85)
Yes, unplanned	0.02 (0.01–0.05)	0.02 (0.01–0.05)
**Had non-consensual sexual intercourse with husband in past 12 months **		
Never	Ref.	Ref.
Occasionally	0.83 (0.56–1.21)	0.78 (0.44–1.39)
Frequently	0.96 (0.56–1.64)	0.48 (0.22–1.04)
**Availability of health-care services in the camp**		
No health-care centre, but field workers visit home	Ref.	Ref.
Yes	1.32 (0.82–2.11)	3.92 (2.01–7.67)

CI: confidence interval; OR: odds ratio; Ref.: reference group.

^a^ Attended no formal educational institutions, although some had attended institutions such as madrasah (a mainly religious and basic education provider).

## Discussion

Our estimation of contraceptive use among Rohingya women is consistent with that for refugees in other areas such as West Bank and Gaza Strip (463/841; 55.05%)^21^ and the Syrian Arab Republic (77/151; 51.0%),^22^ and 2–10 times higher than among refugees in Chad, the Democratic Republic of Congo, Djibouti, Guinea, Mali and Pakistan.^23^^,^^24^

Our study has revealed that around one half of the female Rohingya refugees do not use contraceptives, mainly because of their husbands’ disapproval and their religious beliefs. The scale of the challenge facing family planning advisors is illustrated by the fact that almost all of the women who experienced an unplanned pregnancy within the 2 years before the study were still not using contraceptives.^13^^,^^25^

The Rohingya people have deep-rooted misconceptions regarding the use of contraceptives; indeed, the majority of these people incorrectly believe that the religion of Islam does not permit the use of contraceptives, and that such use may even put women at risk of developing health issues.^3^ In addition, given that they do not have residency status in Bangladesh, many believe that the contraceptives provided are intended to cause irreversible sterilization.^25^ A further challenge is that the Rohingya people often do not have any plans regarding their reproductive lives; they consider children to be a gift from God and want as many as possible.^25^ However, our estimated significant association between women who used contraceptives but also viewed their most recent pregnancy as planned may be indicative of changing attitudes.

Increased autonomy among the women regarding their reproductive lives, increased participation in activities outside their homes and greater access to reproductive health-care services could significantly increase the use of contraceptives and result in fewer unplanned pregnancies, particularly among women who have not received any formal education or who married at a young age.^4^^,^^18^^,^^26^ In addition, increasing awareness of their sexual and reproductive rights may also help to improve the situation,^27^ as has been found in research conducted in other refugee settings.^23^^,^^28^

Our estimate of contraceptive use by Rohingya women is higher than that made in 2018 (757/2227; 33.99%),^3^ a result of a wide range of programmes such as counselling and the provision of free contraceptives offered by various health-care stakeholders working in the Rohingya camps.^3^ However, the majority of the women who participated in our survey had not received the government-recommended six or more visits by family planning field workers.^29^^,^^30^ More work therefore needs to be done to achieve the current international and national targets of ensuring effective contraception for every woman.^6^^,^^31^

Our study revealed that, of the Rohingya women who do use contraceptives, the contraceptive injection or oral contraceptive pill are preferred.^3^ Permanent or long-term methods, such as sterilization and intrauterine devices, respectively, as well as the use of condoms, are uncommon;^25^ however, these are the very contraceptive methods that are often considered to be priorities by health-care providers working in the camps.^3^ Targeted programmes that tackle this potential discrepancy between the preferences of the contraceptive users and the priorities of the health-care providers would therefore appear to be critical in increasing contraceptive use. Community leaders could also play an important role in promoting the use of alternative methods to the contraceptive injection or pill.^32^

The main strength of our study is our primary data set collected from the Rohingya population. However, our study also had several limitations. As our study is based on data from a cross-sectional survey, our findings are correlational only. Moreover, we could not include all potential confounders in the model that may have a significant influence on decisions regarding current contraceptive use, such as the opinions of husbands or past use of contraceptives. Finally, we analysed self-reported data without any scope for validation by the interviewers; our data may therefore be subject to reporting or recall errors. 

To conclude, we recommend further study to obtain more precise information about contraceptive use and its associated barriers and facilitators. We also recommend programmes targeted at women of reproductive age and their husbands, those who have received no formal education, and religious leaders. Finally, the inclusion of women’s husbands in family planning services,^3^ and the integration of maternal and child health-care services with family planning services,^33^ could also help to increase the use of contraceptives. 
